# Self-organizing bioinspired oligothiophene–oligopeptide hybrids

**DOI:** 10.3762/bjnano.2.57

**Published:** 2011-09-05

**Authors:** Alexey K Shaytan, Eva-Kathrin Schillinger, Elena Mena-Osteritz, Sylvia Schmid, Pavel G Khalatur, Peter Bäuerle, Alexei R Khokhlov

**Affiliations:** 1 Institute of Polymer Science, University of Ulm, Albert-Einstein-Allee 47, D-89069 Ulm, Germany; 2 Biology Department, Moscow State University, 119991 Moscow, Russia; 3 Institute of Organic Chemistry II and Advanced Materials, University of Ulm, Albert-Einstein-Allee 11, D-89081 Ulm, Germany; 4 Institute of Organoelement Compounds, Russian Academy of Science, 119991 Moscow, Russia; 5 Physics Department, Moscow State University, 119991 Moscow, Russia

**Keywords:** amyloid-like fibrils, bioinspired conjugates, molecular dynamics simulations, oligopeptides, oligothiophenes, self-assembly

## Abstract

In this minireview, we survey recent advances in the synthesis, characterization, and modeling of new oligothiophene–oligopeptide hybrids capable of forming nanostructured fibrillar aggregates in solution and on solid substrates. Compounds of this class are promising for applications because their self-assembly and stimuli-responsive properties, provided by the peptide moieties combined with the semiconducting properties of the thiophene blocks, can result in novel opportunities for the design of advanced smart materials. These bio-inspired molecular hybrids are experimentally shown to form stable fibrils as visualized by AFM and TEM. While the experimental evidence alone is not sufficient to reveal the exact molecular organization of the fibrils, theoretical approaches based on quantum chemistry calculations and large-scale atomistic molecular dynamics simulations are attempted in an effort to reveal the structure of the fibrils at the nanoscale. Based on the combined theoretical and experimental analysis, the most likely models of fibril formation and aggregation are suggested.

## Introduction

Amyloid and amyloid-like fibrillar aggregates, formed by natural proteins or oligopeptides, have attracted much attention both due to their involvement in medical pathologies (such as Alzheimer’s disease, Parkinson’s disease, etc. [1–3]) and their possible applications as building blocks in nano- and biotechnology. Understanding the molecular details of peptide self-assembly into fibrillar aggregates has been a challenge owing to the large size, low solubility, and the noncrystalline and heterogeneous nature of the fibrils.

During the last decade, considerable progress in our understanding of the principles of fibril formation has been made owing to numerous experimental and theoretical studies and, importantly, the resolution of peptide arrangements at the atomistic level by means of X-ray crystallography and solid-state NMR [4–6]. The outstanding ability of amyloidogenic peptides to self-assemble and form extremely stable and tough nanostructures has been realized, and thus they are now actively probed as building blocks for various nanotechnology applications by covalently binding them to synthetic moieties [7–10], and engineering fusion proteins [11] and colloidal particles [12–13]. However, we are still too far away to say that the complete picture of the fibril self-assembly is now available at the molecular, nano- and microscale levels.

**Figure 1:**
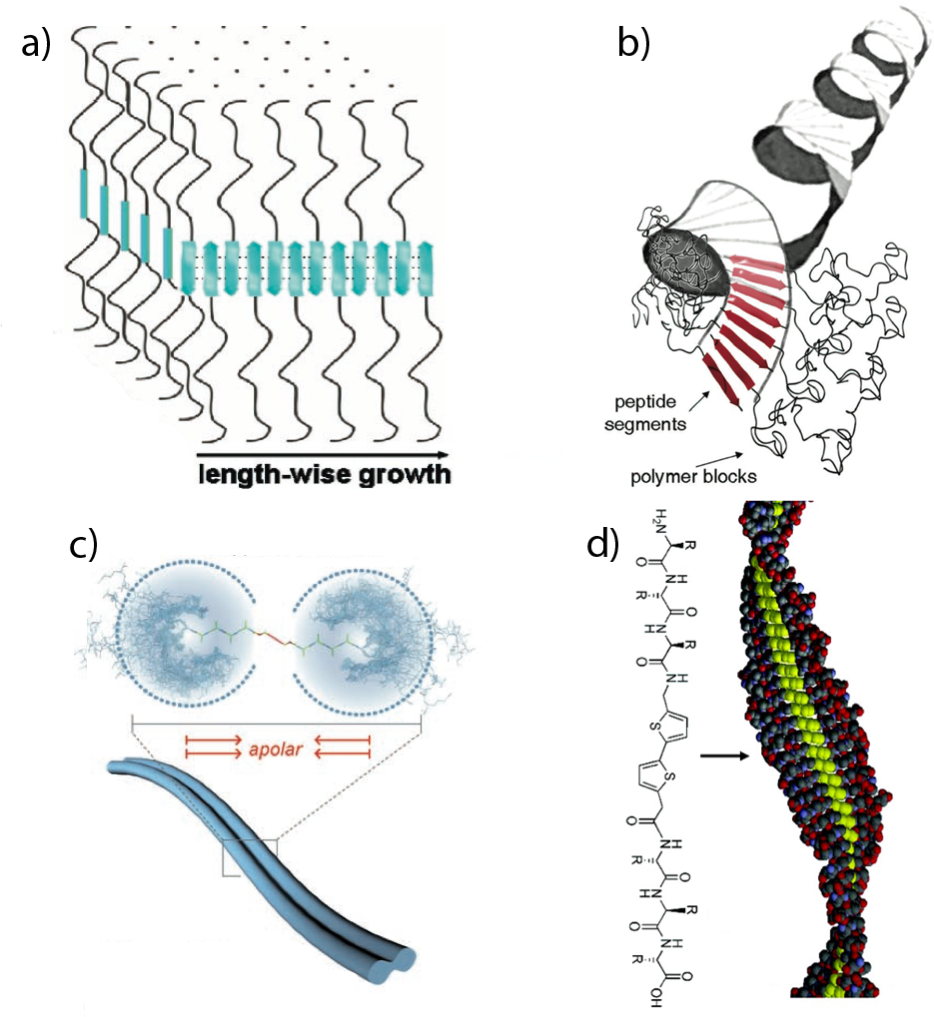
Various morphological organization examples of fibrillar aggregates that can be formed by polymer bioconjugates: (a) Wide planar tapes [18]; (b) helical superstructure [19]; (c) tapes with a dumb-bell shaped cross section [20]; (d) helical tapes with a thiophene block in the middle [21].

In this area, the approach that is gaining more and more attention is the synthetic conjugation of peptides to other molecular compounds. Conjugates of synthetic and natural macromolecules are of great current interest because of their promising biomedical, microelectronic, and other advanced technological applications [7–10,14–15]. Covalent attachment of synthetic polymer blocks to amyloidogenic peptide sequences leads to a new class of block copolymers that can inherit typical properties of their constituents, e.g., enhanced performance characteristics, conductivity, biocompatibility, and high propensity for self-organization. In this respect, oligothiophene–oligopeptide conjugates are of particular interest. The conjugation of oligothiophenes and amyloidogenic peptides may result in new compounds that supplement the potentially semiconducting, optical, and electroluminescent properties of oligo- and polythiophenes with the self-assembling, specific binding, and stimuli responsive behavior of biological moieties, thus opening up opportunities for the design of smart materials at the nanoscale. An example of a hypothetical fibrillar aggregate structure, formed by a bithiophene covalently linked to peptide sequences, is illustrated in Figure 1d.

Several reviews [7–9,15] summarize the recent progress in the field of the chemistry of polymer bioconjugation in which the biconjugation with amyloidogenic peptides is one of the most frequently used techniques. The resulting interplay of intermolecular interactions is affected by both the synthetic and peptide parts, leading to an even greater structural polymorphism than observed in natural amyloid fibers, keeping in mind that synthetic chemistry provides more variability in the structure of the building blocks, including branched molecular topologies [16].

A number of hypothesized self-organized morphologies that may be adopted by various polymer bioconjugates are shown in Figure 1. Moreover, the interplay between different interactions may also suggest a dependence of the supramolecular organization on the external conditions, such as temperature, solvent quality, pH value, etc. [17].

While the chemical structure of the aggregating compounds is almost always known, and the fibrillar morphology at the submicrometer scale is resolved by electron or atomic force microscopy, the structure of the fibrils at the atomistic and nanoscales, including the packing of the single molecules, very often remains beyond the capabilities of experimental measurements to elucidate. In particular cases, it becomes possible to gain insight into the intrinsic structure of the fibrils, e.g., by means of X-ray diffraction when the corresponding microcrystals can be obtained, or when sufficient solid state NMR data is available. However, for the majority of compounds and moreover for polymer-bioconjugates, the available experimental evidence regarding the intermolecular interactions is in most cases limited to spectroscopic analysis (IR, UV–vis, CD spectroscopy) and diffraction patterns (X-ray, SAED) and thus the exact structural arrangement at the nanoscale and its connection to the morphology remains elusive.

The molecular simulation methods in this respect become an attractive tool to supplement and interpret the experimental data, because they can fill in the missing gaps in the understanding of the relationship between the structural arrangement and the fibrillar morphology. When 3-D atomistic structures of microcrystals or oligomeric aggregates are available they may be used to construct computational models of the fibrillar aggregates, and through the application of atomistic molecular dynamics (MD) simulations the structural data can be extended into the dynamic domain. Since microcrystals usually only provide the structures of the basic aggregation units, different arrangements of these units into the protofilaments and then fibers may be probed in computer simulations. However, when no initial 3-D structural data is available, the application of computer simulations may be less straightforward. In this case, it is necessary to predict or suggest various trial arrangements, construct the aggregates, and then test their characteristics against available experimental data. The latter approach has a much wider applicability in terms of studied compounds and potential applications for the rational computer-aided design of new macromolecular systems with specific properties.

In this minireview, we discuss the recent progress in the design, synthesis and in computer simulations of new oligothiophene–oligopeptide conjugates that can self-assemble to form nanoscale fibrillar aggregates. The main focus is on the experimental and theoretical results obtained in our group.

## Review

### Synthesis of oligothiophene–oligopeptide hybrids

**Scheme 1:**
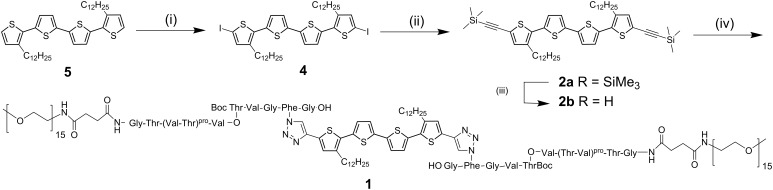
Synthesis of quaterthiophene-β-sheet-peptide hybrid **1** [22]; (i) Hg(II)OAc_2_, CHCl_3_, 0 °C → r.t., 14 h; I_2_, 0 °C → r.t., 6 h; (ii) Cu(I)I, TMSA, Pd(PPh_3_)_2_Cl_2_, piperidine, 60 °C, 2 h; (iii) 6 equiv CsF, MeOH/THF, r.t., 2 h; (iv) 0.4 equiv [Cu(CH_3_CN)_4_]PF_6_, 0.4 equiv Cu(0), DCM, r.t., 40 h.

**Scheme 2:**
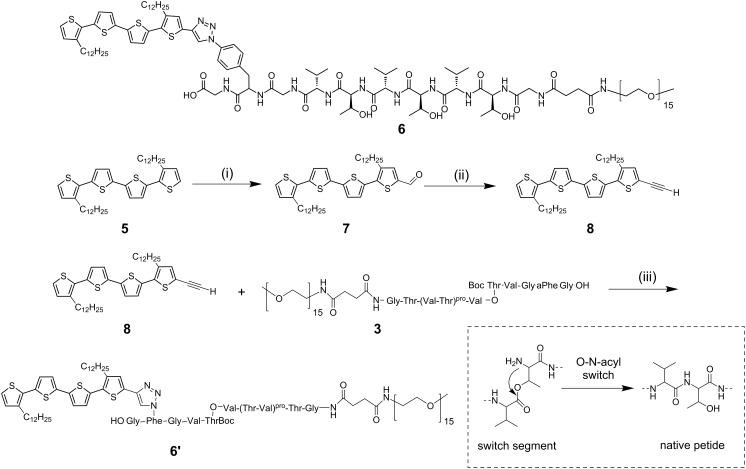
Synthesis of quaterthiophene-β-sheet-peptide hybrid **6** [23]; (i) POCl_3_, DMF, dichloroethane, reflux, 3 h; (ii) K_2_CO_3_, methanol/THF, r.t., overnight; (iii) 0.4 equiv [Cu(CH_3_CN)_4_]PF_6_, 0.4 equiv Cu(0), DCM, r.t., 40 h. (Thr–Val)^pro^ refers to a pseudoprolien unit, (Val–O–Thr) refers to the switch segment (framed bottom right) , and aPhe stands for *p*-azido-Phe.

The hybrid molecules discussed in this paper are bio-functionalized organic semiconductors. They represent either di- or mono-substituted conjugates with a quaterthiophene block and an oligopeptide block containing three repeat units of L-valine-L-threonine. These molecular hybrids – the A–B–A-type compound **1** [22] and the A–B-type compound **6** [23] – and their synthesis are presented in Scheme 1 and Scheme 2, respectively. The peptide blocks were equipped at the termini with poly(ethylene oxide) (PEO) chain. Due to their amphiphilic character and the defined secondary structure of the biological moiety, hybrids **1** and **6** are expected to reveal interesting self-assembly behavior in solution and on solid substrates.

Symmetrically substituted didodecyl-quaterthiophene **5** was chosen as the basic building block (Scheme 1), representing a planar fully conjugated backbone with the ability to self-assemble at the liquid–solid interface into very regular lamellar structures [24]. The intermolecular forces involved are primary van der Waals interactions of the interdigitating long alkyl side chains. Furthermore, the planar conjugated thiophene backbone is well known to interact by π–π stacking to form larger crystalline structures. With respect to the peptide part, the L-valine-L-threonine (Val–Thr)_3_ sequence effectively forms β-sheet secondary structures. Hybrids **1** and **6** were shielded laterally by poly(ethylene oxide) chains in order to enhance solubility and processability and to induce the formation of isolated fibrillar structures.

The synthesis of the semiconductor block, bisethynylated quaterthiophene **2b** and the final oligothiophene–peptide hybrid **1** is shown in Scheme 1: The diiodinated quaterthiophene **4** was synthesized from the corresponding parent compound **5** by iodination at the terminal α-positions, with mercury(II)acetate and elemental iodine in dry chloroform, to give compound **4** in 94% yield.

The remarkable tendency of these peptide blocks to form β-sheets enormously hinders the synthesis of the hybrids due to aggregation during reaction [16,25]. In order to overcome this problem, the aggregation tendency was temporarily suppressed through a synthetic strategy, which employs pseudoprolines ((Thr–Val)^pro^) [26] and a switch ester segment [27] (Scheme 2). The pseudoprolines were inserted as a transient structure-disrupting protecting group for threonine and the switch ester segment as a temporary structural defect in the peptide block. Whereas the pseudoproline unit is removed during standard acidic deprotection conditions, the switch ester segment is preserved at low pH. Reestablishment of the native α-amide peptide backbone can be achieved by an increase in the pH to neutral or even slightly basic conditions.

The peptide segment was obtained by a semi-automated solid-phase supported peptide synthesis (SPPS) by sequential coupling of standard fluorenylmethoxycarbonyl (Fmoc)-protected amino acids and the pseudoproline unit onto a preloaded TentaGel resin [27]. Incorporation of the Fmoc-protected azido-phenylalanine (aPhe) and the addition of the switch ester segment between valine (Val) and threonine (Thr) were accomplished by means of bench top coupling techniques [27]. An end-functionalized poly(ethylene oxide) block, containing 14 or 15 repeat units, was attached to the N-terminus of the supported peptide. Subsequent liberation of the peptide-PEO_15_ conjugate from the support, followed by precipitation, centrifugation and lyophilization, gave the fully protected peptide-PEO_15_ conjugate **3** in 61% yield (Scheme 2) [19].

The PEO–peptide–quaterthiophene hybrid (A–B) **6** was prepared analogously to the A–B–A-type hybrid **1** by the reaction of monoethynylated quaterthiophene **8** and protected peptide–PEO_15_ conjugate **3** [28] (Scheme 2). Ligation of freshly prepared **8** with the PEO-peptide conjugate **3** was accomplished by Cu(I)-catalyzed Huisgen cycloaddition.

### Self-assembly of oligothiophene–oligopeptide hybrids

The self-assembly properties of the A–B–A-type molecules on surfaces were analyzed by means of atomic force microscopy (AFM) as a powerful tool to visualize superstructures in the nano- and micrometer regime [22–23]. The substrate employed was muscovite mica, and tapping mode was chosen for scanning.

**Figure 2:**
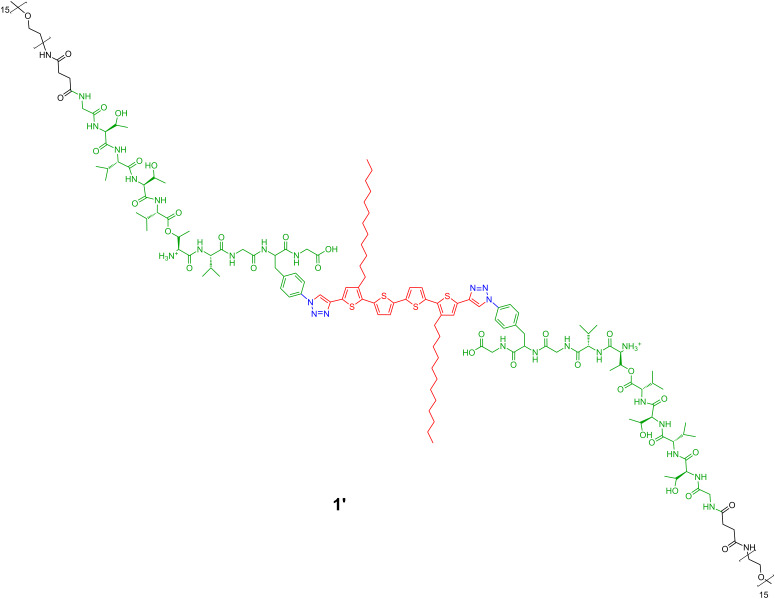
The A–B–A-type hybrid **1** in the deprotected, but still kinked, form **1'**.

For deprotection, the triblock oligothiophene–oligopeptide compound **1** (Scheme 1) was first treated with 30% trifluoro acetic acid (TFA) in dichloromethane. Under these acidic conditions, all protecting groups present in hybrid **1** (*t*-Boc, pseudoproline, *t*-butyl ester) are removed, except for the switch ester segment, which is preserved in the new form **1'**. Thus, molecule **1'** still exhibits a kink in the peptide backbone, as shown in Figure 2.

To investigate the self-assembly of **1'**, several solvents were employed, including water (pH ≈ 4–4.5), aqueous phosphate buffer (pH ≈ 6–6.5), and dichloromethane with two droplets of THF (pH ≈ 2). These attempts resulted in the formation of clusters or irregularly twisted short fibers of **1'** on the mica substrate.

**Figure 3:**
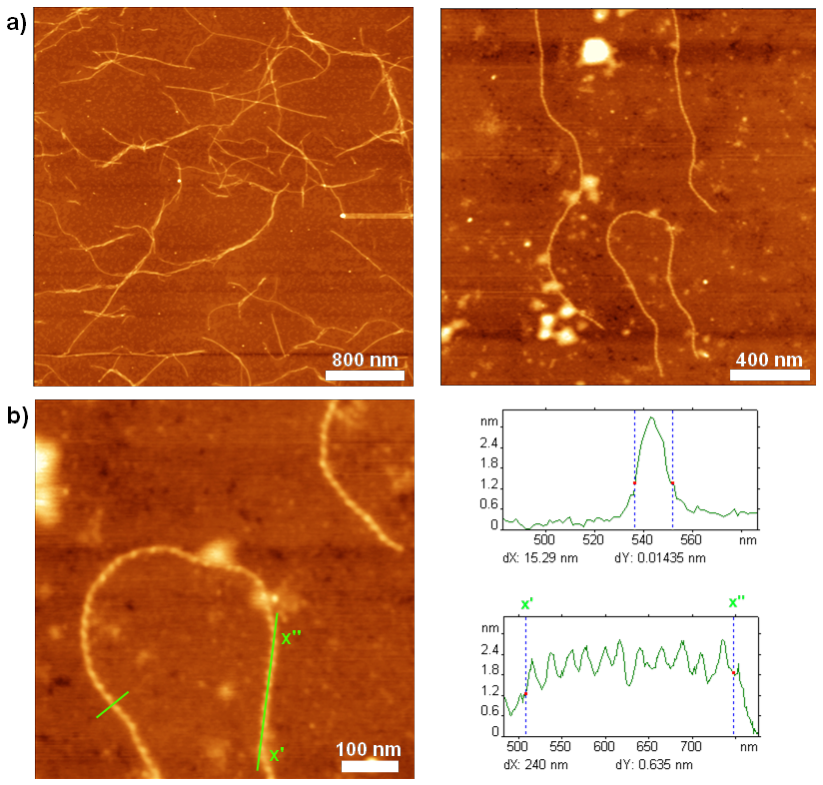
AFM height images of hybrid **1'** on mica from a 1:1 DCM/MeOH solution; a) left: Network of fibers after 2 d; right: Fibrillar features found after 2 h; b) left: Left-handed helical fiber, right: Cross section and height profile from the marked sections in 2b [22].

Due to the hybrid nature of **1'**, two opposing intermolecular interactions could dominate the self-assembly process, namely H-bonding and π–π stacking. Thus, a solvent-guided strategy was employed in order to gain control over the self-assembly process. Deprotected, but still kinked, compound **1'** was dissolved in dichloromethane (a good solvent for the oligothiophene moiety), and to this solution methanol (non-solvent for the oligothiophene part) was added gradually through a syringe pump until a ratio of DCM/MeOH of 1:1 was reached (pH ≈ 5). The switch segments in the peptide moieties were still intact [19,27]. The solution was spin-coated on the mica substrate and well-defined microstructures were visualized by means of AFM (Figure 3).

The fibrillar structures exhibit single object widths of about 12 ± 1 nm (not tip corrected), with height maxima of 3 ± 0.4 nm, and lengths of up to several micrometers. The presence of these fibers was confirmed in our recent work [22] by means of transmission electron microscopy (TEM). The finding of helical self-assembled fibers for compound **1'** (Figure 3b) is rather surprising, since the efficiency of the switch ester segment in suppressing β-sheet formation had been demonstrated previously [19,25].

**Figure 4:**
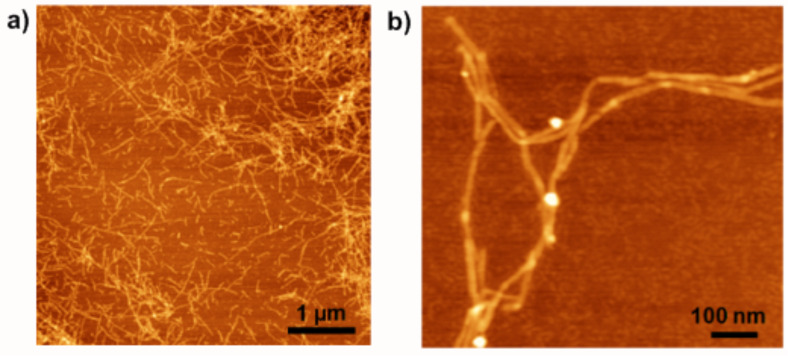
AFM images of the switched PEO–peptide–quaterthiophene–peptide–PEO compound **1** [22].

In order to re-establishing the native peptide backbone of **1**, 0.001 M sodium hydroxide dissolved in methanol was added to a solution of **1'** in dichloromethane. The AFM images (Figure 4) showed fibrous structures with lengths of up to 1–2 μm, heights of 2.4 ± 0.4 nm and widths of 11 ± 2 nm. The lack of helicity in the fibers (at least at our resolution capacity) indicated that in this state of the peptide the fibers do not possess a chiral substructure. This finding was confirmed by means of TEM [22].

**Figure 5:**
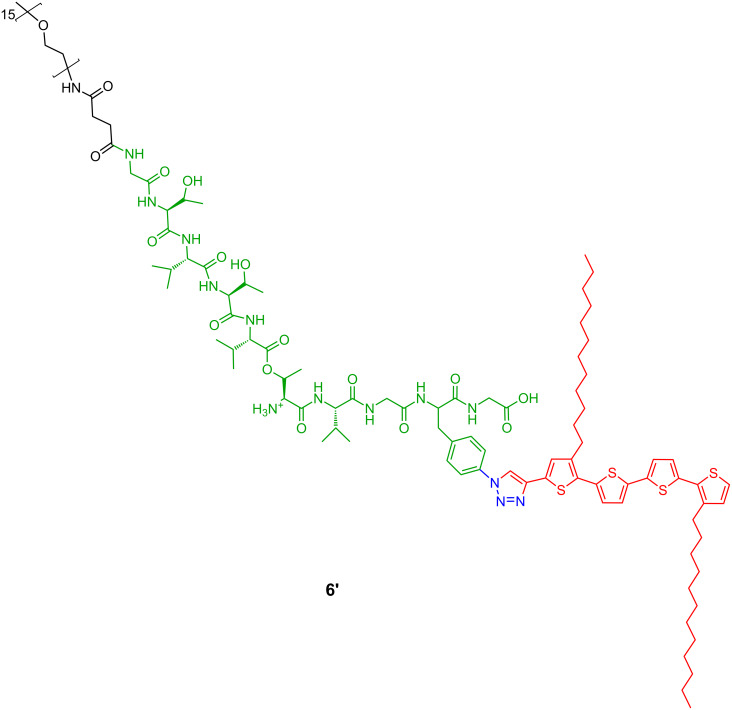
A–B system **6'** in deprotected, but still kinked, form.

The self-assembly of the unsymmetrical mono-β-sheet-peptide–oligothiophene hybrid **6** (Scheme 2) was investigated on mica substrates by AFM as well. For deprotection, this compound was treated with 30% trifluoro acetic acid (TFA) in dichloromethane. Under these acidic conditions, all protecting groups present in hybrid **6** are removed, except for the switch ester segment, which is preserved. Thus, the molecule **6'** still exhibits a kink in the peptide backbone (Figure 5).

**Figure 6:**
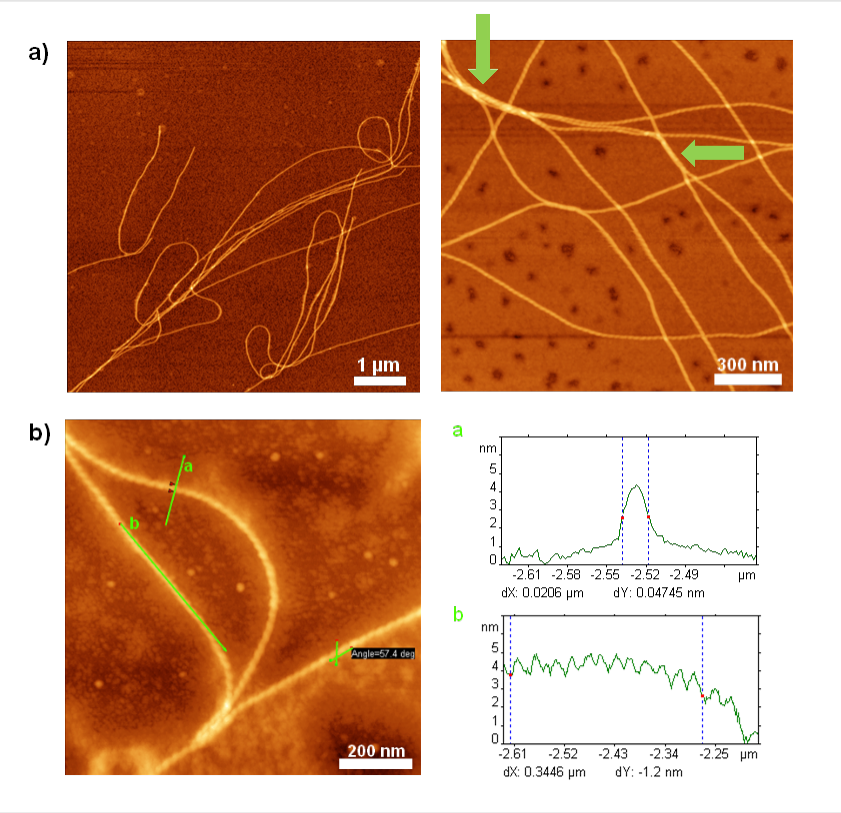
AFM height images of **6'** on mica from a 1:1 DCM/MeOH solution. a) left: Image of fibers obtained after 2 d; right: Zoom of fiber networks; green arrows: Contact points of individual fibers; b) left: Zoom of left-handed helical fibers; right: Cross section and height profile of helical fiber [22].

Taking into account the experimental findings described before for the disubstituted A–B–A system **1'**, similar conditions were chosen for A–B system **6'**. The 1:1 DCM/MeOH solution of **6'** was established much faster than the one of **1'** in order to form the highly regular, exclusively left-handed, helical fibers, which can be observed in AFM (Figure 6).

The fibrillar structures show single object widths of about 20 ± 2 nm (AFM, not tip corrected), height maxima of 3.5 ± 0.4 nm, and lengths of up to several micrometers (Figure 6a, left). A tightly wound, strictly left-handed substructure was resolved for the fibers, possessing a pitch length of 25 ± 2 nm (Figure 6b, right). At several points, contact between the individual fibers can be seen (green arrows in Figure 6a, right) but there is no evidence for fiber intertwining to form multihelices.

**Figure 7:**
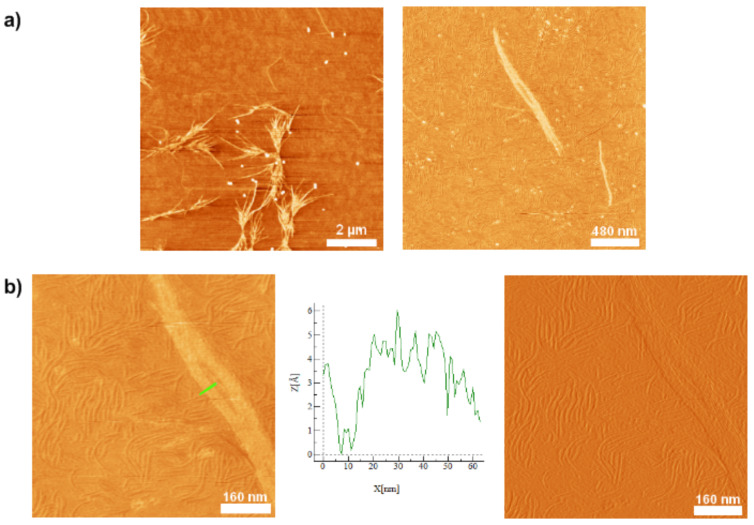
AFM height and amplitude images of fully switched PEO–peptide–quaterthiophene **6** on mica [23]. a) left: AFM height image obtained after 7 d; right: Zoom of single fiber; b) left: Height image of single fiber consisting of smaller filaments; middle: Cross section corresponding to the marked section in b), right: Amplitude image of b) left.

Based on the results obtained from the symmetric system **1'**, the same controlled way of transferring the switch ester segment into native amide bonds was chosen for **6'**. The AFM investigations of **6** adsorbed onto the mica substrate revealed fibrous structures (Figure 7).

The pattern of the self-assembled fibers, though, does not correspond to a network but is instead more reminiscent of bundles or clusters of fibers (Figure 7a, left). Surrounding these larger areas of concentrated material, single fibers can be found (Figure 7a). At higher resolution it was shown that the “single” fibers consist of several smaller filaments aligned in parallel (Figure 7b), which explains the irregularly frayed appearance of the bigger fibers. The cross-section analysis reveals, as well, the structure of the filaments composing the bigger fiber (Figure 7b, middle). Especially in the close view images (Figure 7b, left: Height, right: Amplitude), it can clearly be seen that shorter fibrillar structures are also deposited as singular features on the mica surface. These features can be considered to represent single filaments that have not been self-assembled into the bigger structures (“fibers” or bundles).

Because of the composition of the bigger fibers from filaments, it was impossible to determine unambiguous values for the height and width of the bigger fibers or even bundles. For the bigger fibers, lengths between 1 and 2 µm and heights of 1 ± 0.2 nm were observed. Widths ranged mostly between 15 and 48 nm but also sporadic widths of up to ≈80 nm could be found. In order to gain more information on the smallest objects observed, the filaments seen in the background of the images were investigated with respect to their geometry as well. They showed lengths of 100–200 nm, widths of 6–8 (±2) nm and heights of 0.4–0.7 (±0.2) nm.

Compared to the fibers obtained in the kinked state of the molecule, **6'** (Figure 6), the microstructures found for the fully stretched out peptide, **6**, seem to be, by far, less self-contained and persistent (Figure 7). Not only do they lack the left-handed helical superstructure observed for the latter, but also the filaments containing molecules **6** seem to be more prone to self-assemble into higher structures (fibers and bundles), whereas the helical fibers of the hybrid **6'**, are more insulated and do not seem to merge into any higher order (Figure 6). In addition, fibers from **6'** observed by AFM are much longer and well confined. These observations lead to the assumption that the mode of self-assembly of the individual molecules in their respective states, kinked **6'** and stretched **6**, differs profoundly. Whereas the kinked hybrids, **6'**, seem to self-assemble in such a way that the intermolecular noncovalent interacting forces are fully saturated, i.e., the formed microstructures do not make use of any unsaturated sites for, e.g., hydrogen bonding, the stretched out state of the peptide in **6** seems to form higher structures, in which unsaturated sites for intermolecular interactions may be present.

### Models of molecular aggregates

Molecular models were developed and simulated on the basis of our experimental findings. Quantum chemical calculations based on the Austin Model 1 (AM1) level were performed on isolated, and bundles of, oligothiophene–oligopeptide molecules for the case of **1'**. The PEO units were omitted in the calculations for simplification. The models were constructed in vacuum.

**Figure 8:**
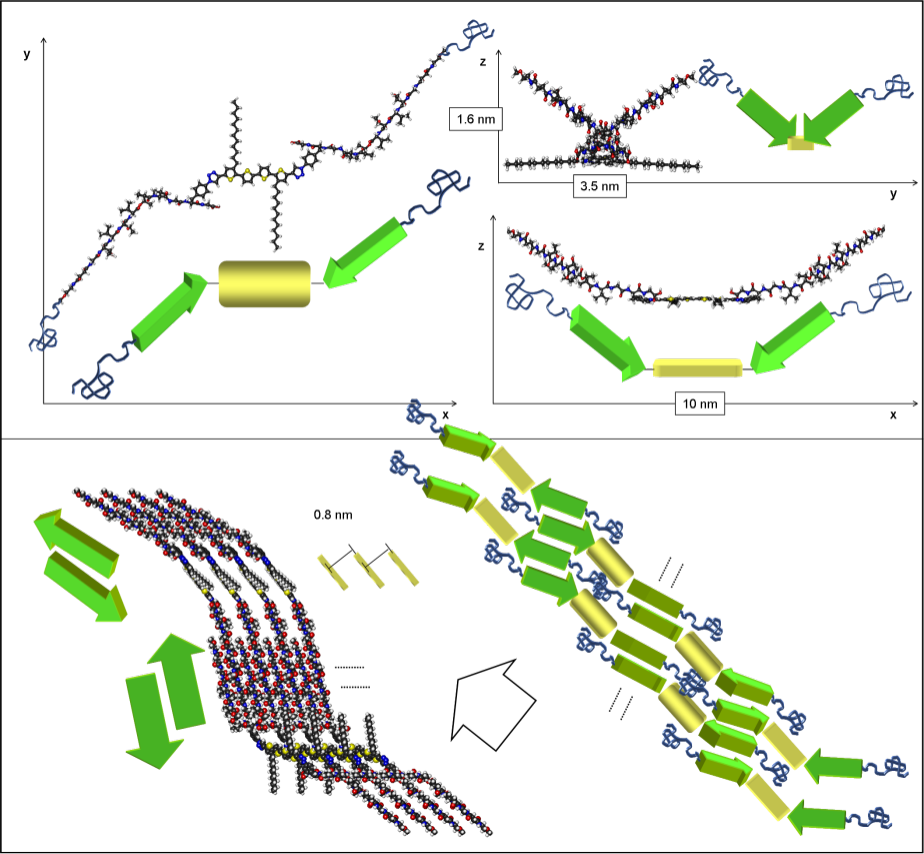
Calculated minimum energy conformation of oligothiophene–oligopeptide hybrid **1'** in the three Cartesian directions (top). Calculated model of an ensemble of eight molecules (bottom, left) as well as a model cartoon (bottom, right). White arrow shows the fiber growth direction [22].

Accounting for the unexpected formation of helical fibers in the kinked state of hybrid **1'**, as discussed in the previous section (see also [22]), we suggested the molecular models shown in Figure 8.

The symmetric optimized conformation of **1'** was employed as the basis for the development of a model. The slightly offset packing of two **1'** molecules leads to 8 favorable H-bond interactions in each peptide part. Thus, the peptide strands interact in a favorable antiparallel fashion (green arrows in Figure 8). The oligothiophene backbones (yellow boxes in Figure 8) are separated by 8 Å, avoiding an efficient π–π interaction in the **1'** fibers. The voids in between the oligothiophenes can be filled by the nondepicted flexible PEO-chains (blue coils in Figure 8).

This model also accounts for an inherent left-handed superstructure of the formed fibers, arising from the function of the oligothiophene block as a rigid and preorientating spacer between the peptide arms of **1'**. In addition, the antiparallel arrangement of the peptide blocks stabilizes the fiber in a helical manner and is responsible for fiber growth. The height of the superstructures is predicted to be 3.2 nm, fitting well with the experimental values of 3 ± 0.4 nm. The calculated angle between the pitch and the fiber growth direction (45 ± 2°) corresponds well to the experimental finding (40 ± 5°). The model predicts three hybrids of **1'** to be needed for the completion of one loop, leading to a theoretical pitch length of 18 ± 1 nm, which is in agreement with the experimental finding from AFM images (20 ± 1 nm). The thicknesses of the fibers experimentally determined by AFM and TEM correspond to the lateral interaction of 8–12 molecules of **1'** in the theoretical model.

**Figure 9:**
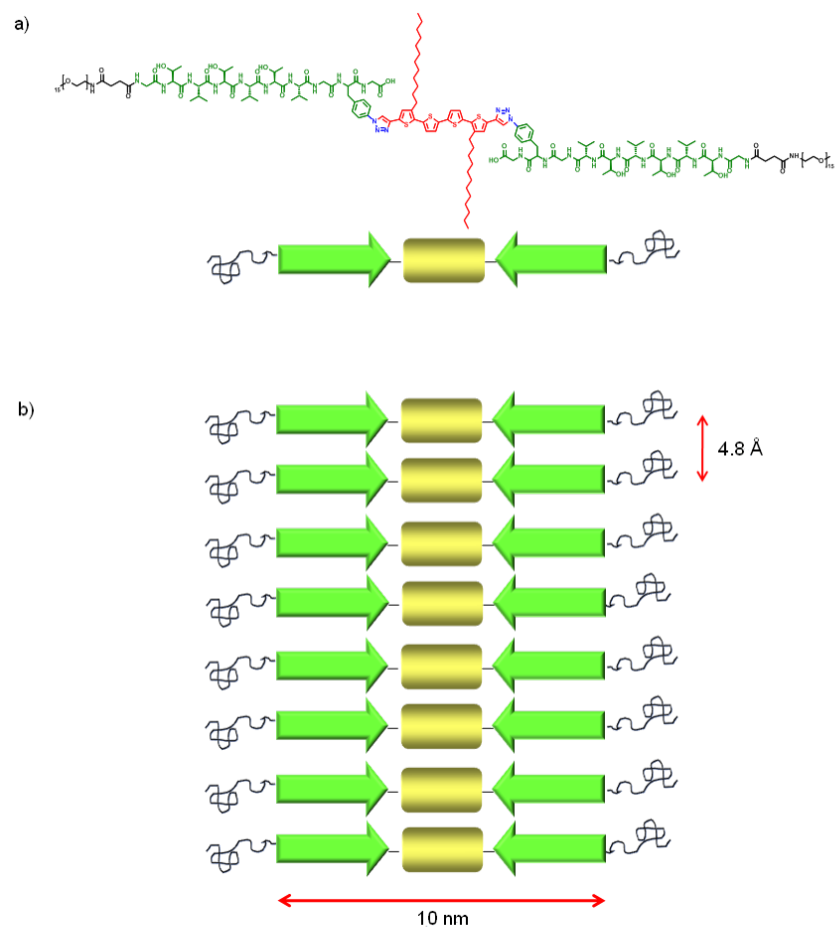
a) Schematic representation of hybrid **1**. Black coils: PEO chains, green arrow: Peptide strand; yellow box: Quaterthiophene; b) Model of parallel β-sheet superstructures of hybrid **1**.

For the native state of the peptide moiety in hybrid **1**, the following model for the superstructure can be deduced from the stretched molecular geometry and by following the best packing taking into account the intermolecular interactions observed in the calculated model of **1'** (Figure 9).

The fibers observed in AFM and TEM do not display any helical superstructure. Hence, a flat arrangement of the single molecules in a β-sheet structure is most likely. The β-sheet structure itself was corroborated by IR spectroscopy and SAED (*d* spacing of 4.8 ± 0.1 Å). The calculated molecular length of **1** (without the flexible PEO chains) amounts to 10 nm, which fits very well with the experimentally observed widths of the fiber of 11 ± 2 nm (AFM). The height of the fibers observed on the mica was 2.4 ± 0.4 nm. It is known, that the height of a single layer β-sheet with this peptide sequence is 0.9 ± 0.1 nm, so a double layer structure of two β-sheets on top of each other is assumed based on the data available. Such multilayer β-sheets are well known in literature and result from interactions of the hydrophilic or hydrophobic sides, respectively, of two β-sheets [20]. In such a manner, unfavorable interactions with the solvent can be prevented.

**Figure 10:**
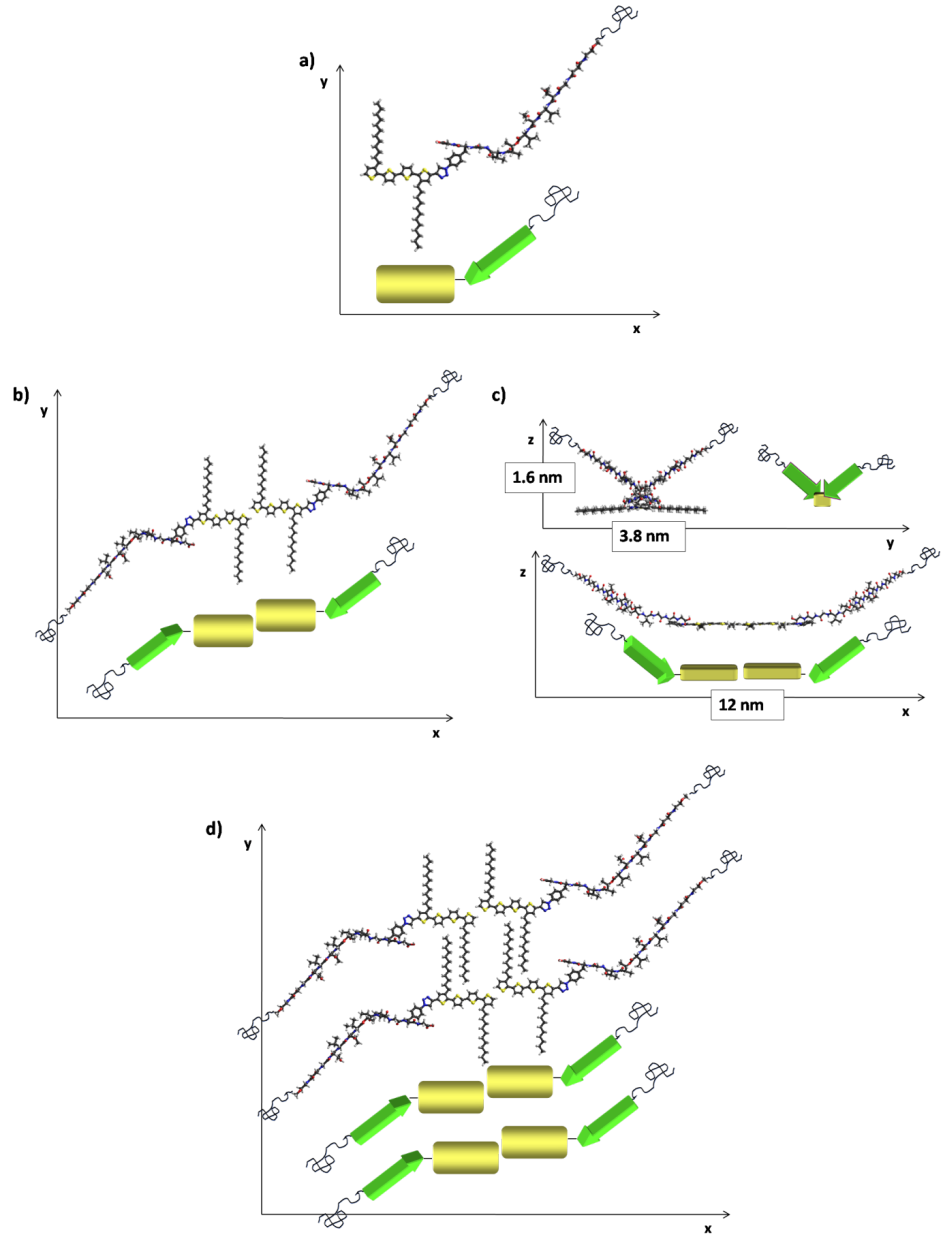
Model for the self-assembly of hybrid **6'**, based on the theoretically calculated conformation of **6'** and the model for hybrid **1'**; a) conformation of **6'**; b) and c) depiction of dimers of **6'** in the three Cartesian directions; c) proposed ensemble of four molecules of **6'**.

The model for the self-assembly of **6'** is depicted in Figure 10. The superstructure was deduced from the theoretically calculated molecular geometry of **6'** and by taking into account the knowledge of the intermolecular interactions of the thiophene moieties and the calculated model of **1'**.

The representation of the molecule’s minimum energy conformation and its schematic representation is given by the same code as that used for the model of **1'** (yellow box: Quaterthiophene backbone; green arrow: Peptide segment; blue coils: PEO chains). When forming dimers from molecules of **6'** (kinked state), such as depicted in Figure 7b and c, an analog of hybrid **1'** (referring to the arrangement of the peptide arms with respect to the conjugated backbones) is obtained. Thus, such dimers should mimic the single molecules of the corresponding disubstituted hybrid **1'** with respect to the formation of inherently left-handed helical fibers (Figure 8, bottom panel). The driving forces for the formation of such proposed dimers could be the compensation of dipole moments of the single molecules in addition to a favorable intermolecular interaction of hydrophilic (peptide–PEO) and hydrophobic (quaterthiophene) parts of the molecules.

Furthermore, the model revealed a separation of 8 Å between the quaterthiophene backbones of two adjacent dimers of **6'**, due to the antiparallel intermolecular interaction of their peptide arms, similar to the case of **1'** (Figure 8). In the case of the postulated formation of dimers of **6'**, however, favorable van der Waals interactions of the alkyl side chains are also involved (Figure 10d) [29]. Eventually, the voids in between laterally adjacent molecules can be filled by the PEO-chains due to their high degree of flexibility, as in the case of **1'**.

The dimensions of the left-handed helical fiber observed in AFM for hybrid **6'** would fit well with such a proposed model. The experimentally observed fibers of **6'** are wider than for hybrid **1'** (20 ± 2 nm versus 11 ± 2 nm). Thus, more dimers of **6'** interact laterally in the superstructure, possibly due to favorable van der Waals interactions of the alkyl chains, leading to a greater width of the fiber. The height of the fiber was experimentally determined to be 3.5 ± 0.4 nm for hybrid **6'**, which is comparable to the height determined for hybrid **1'** (3 ± 0.4 nm). Hence, also the observed angle between the pitch and the fiber growth direction, which was determined to be 55 ± 3° for **6'** as opposed to 45 ± 2° for **1'**, becomes comprehensible, since for a larger number of laterally interacting molecules, and a higher resulting width of the fiber, a more obtuse angle between the pitch and the fiber growth direction must result, if the same overall height of the fiber for **1'** and **6'** is to be maintained (Figure 8). Eventually, for hybrid **1'**, it was predicted that three hybrids are needed to complete one loop of the helical structure resulting in an experimental pitch length of 20 ± 1 nm (see above). The length of a single molecule of **1'** amounts to 10 nm, whereas the length of a dimer of **6'** is approximately 12 nm. Thus, the greater pitch length of **6'** (25 ± 2 nm) fits very well with the proposed model based on the assumption that also for **6'** three dimers are needed to complete one loop of the helix.

### Atomistic molecular dynamics simulation

To further enhance our theoretical understanding of the principles governing the formation of the fibrillar structures from thiophene–peptide conjugates and to gain more insight into the structure and dynamical behavior of the aggregates at finite temperatures, a theoretical methodology based on classical mechanical force fields and molecular dynamics simulations was developed [23]. Although molecular models based on classical mechanics lack the level of precision in describing intermolecular interactions when compared to quantum chemistry models, they allow much higher levels of conformational space sampling, which are needed to reveal the optimal conformation of the aggregates and incorporate the entropic finite temperature effects, which may lead, for instance, to the twist of the fibrils induced by an increase in backbone dynamics [30]. Computer simulations provide an important opportunity to shed light on the possible supramolecular organization patterns, their stability and the governing interplay of intermolecular interactions. To the best of our knowledge, we were the first to apply the rational principles of structure prediction by means of conformation space search based on molecular mechanics, and subsequent MD simulations to study the peptide-directed, noncovalent assembly of thiophene-peptide hybrids [23,31–35].

However, even in classical MD simulations, the rate of conformational sampling is not enough to observe spontaneous formation of fibrillar aggregates; hence, a special computational methodology incorporating the available experimental evidence was developed.

**Figure 11:**
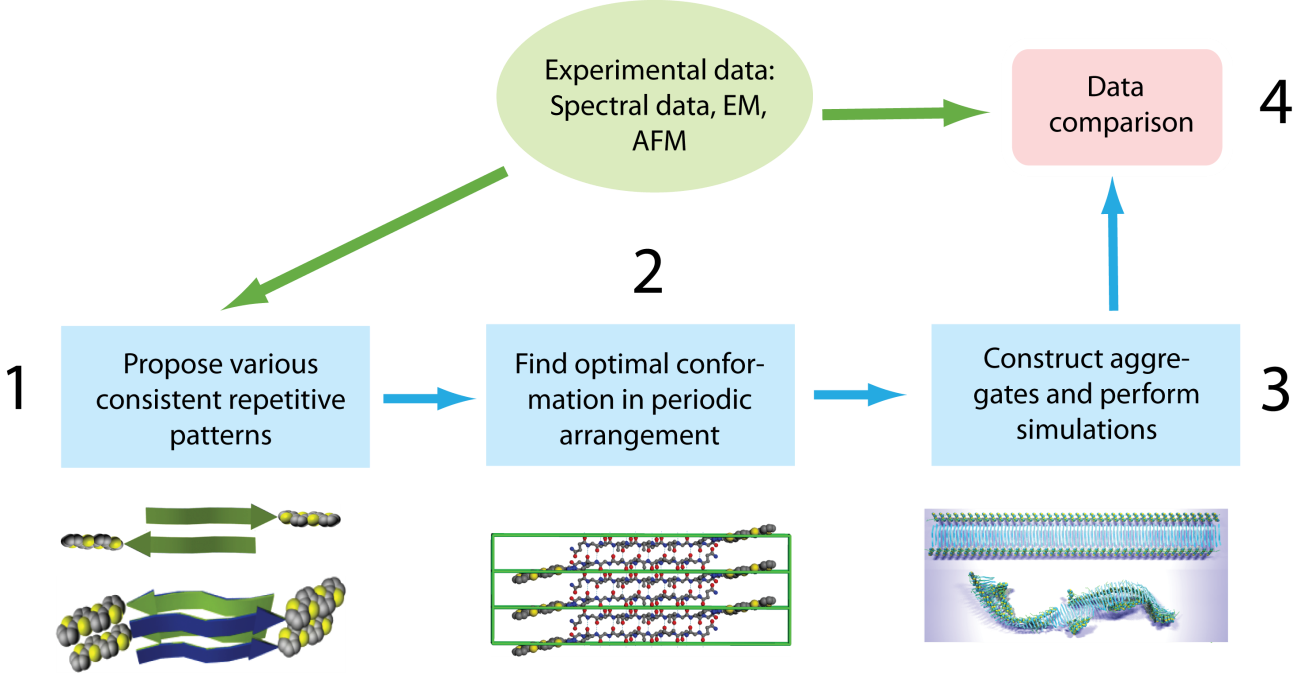
Theoretical analysis workflow (see text).

As a starting point for the theoretical considerations of the experimentally observed nanofibers, the molecular structure of the molecule was constructed. Our theoretical research methodology consists of four main steps schematically depicted in Figure 11.

First, the available experimental data, including available X-ray structural data, for the arrangement of peptide moieties in biological amyloid-like fibrils was analyzed, and possible periodic arrangements of molecules consistent with experimental results were proposed. Second, an approach based on MD simulations was applied to obtain periodic molecular arrangements that correspond to the local free-energy minimum where all local degrees of freedom (torsion angles, side chain conformations, hydrogen bonds) achieve their optimal positions. Then, these optimal arrangements were used to construct long fibrillar aggregates, the dynamic and statistical behaviors of which were investigated by means of MD simulations. Finally, the results obtained from analysis of the simulations were compared with experimental data, which enables us to suggest the most likely molecular arrangement pattern that should be observed in the experiment.

We demonstrate this approach through the example of the A–B system **6** in the native state of the peptide. In principle both parts of this hybrid molecule (thiophene part and peptide part) may be capable of strong intermolecular interactions, thus leading to the formation of highly anisotropic structures such as nanofibers, nanorods, etc. However, the IR spectroscopic data for the compound under study revealed that the β-sheet structure formation was at least involved in the self-assembly mechanism of our nanofibers. While the formation of fibers at the nanometer scale (especially amyloid-like fibers) from separate peptide moieties due to β-sheet aggregation is a common self-assembly mechanism, as discussed previously, it is natural to assume that similar peptide–peptide interactions play the structure-determining role during the aggregation of our hybrid compound and form the scaffold of the fibrils.

Relying on the basic ideas and our current understanding of amyloid fibril aggregation patterns [2,4,36], one can hypothesize that the basic arrangement pattern of peptides in the fibrils is either simply single layer β-sheets (as is sometimes seen for peptide polymer conjugates [19–20]) or double layer aggregates (as seen in many natural fibrils). One can propose two basic periodic arrangements of hybrid molecules in single layer tapes based on an either parallel or antiparallel organization of the peptide moieties in the β-sheets. Several other double layer arrangements are then derived from these single layer arrangements by stacking the β-sheets face-to-face in an aggregation manner similar to the cross-β-spine structure of amyloid fibrils (for more detail, see, e.g., [37]). Below we present the details of the periodic arrangement construction by using the combination of molecular alignment and MD simulations.

**Figure 12:**
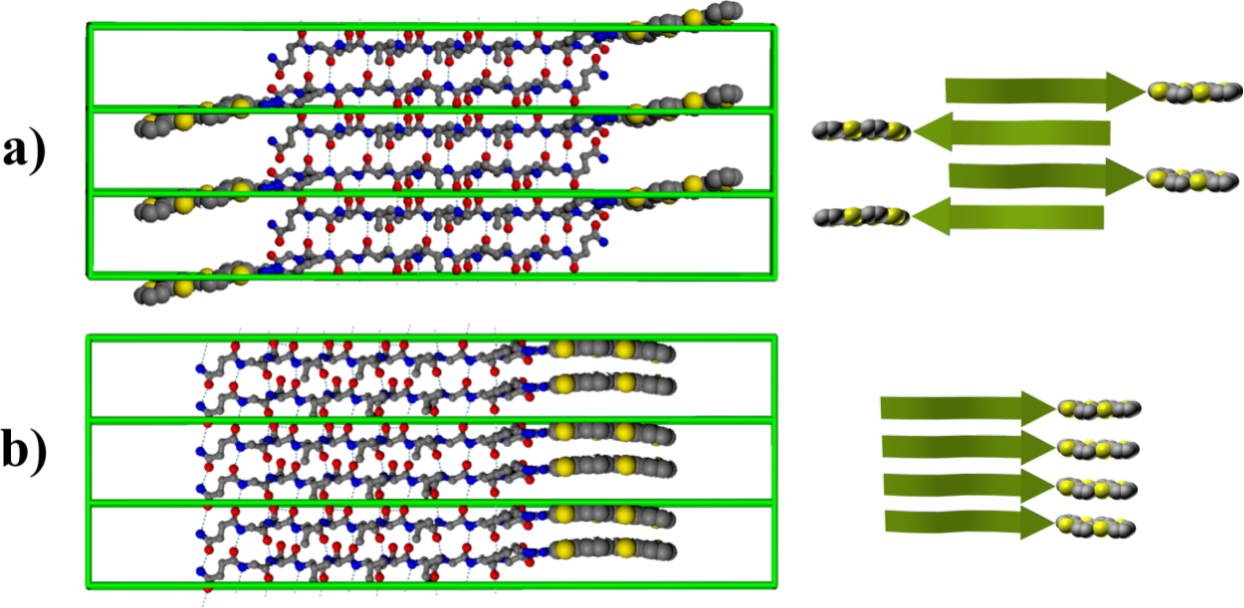
Constructed periodic crystalline cells for (a) antiparallel and (b) parallel arrangement of peptide strands in single-layer fibrils. Alkyl chains are not shown. Dashed lines represent the hydrogen bonds responsible for β-sheet formation. Right insets present the principal arrangement of peptide and thiophene moieties [23].

The construction of aggregates from single molecules can be done with the inclusion of subsequent minimization and relaxation steps as follows. First, the conformations of the initial molecules were adjusted: The peptide block is considered to be in the form of a β-strand engaged in either an ideal parallel or ideal antiparallel β-sheet; this is solely determined by the values of the dihedral 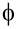 and ψ angles of the peptide backbone, which are known to be 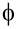 = −119°, ψ = 113° for β-strands engaged in parallel β-sheets and 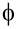 = −139°, ψ = 135° for β-strands engaged in antiparallel β-sheets. The thiophene block, including the 4-azidophenyl-alanine side chain and alkyl chains, is considered to be in a planar, extended conformation corresponding to the local energy minimum. In order to construct a proper periodic arrangement that could be used as an elementary structure to construct long fibrils, a corresponding periodic unit cell that consists of two (for single-layer fibrils) or four (for double-layer fibrils) molecules was derived. The period implied by the unit cell along the fibrillar axis was set to be 4.8 Å per β-strand so as to correspond to that generally observed in amyloid fibrils (9.6 Å for two strands). As a first step, two molecules were arranged into two single-layered periodic structures with a parallel and an antiparallel arrangement of β-strands, the peptide segments in both cases were aligned in register so as to maximize the number of interstrand hydrogen bonds, which was monitored during the system arrangement (Figure 12). Energy minimization for such periodic single-layer systems (that can be regarded as infinitely long crystalline tapes) was then performed followed by a long relaxation MD run.

The single-layer periodic structures obtained were then used as building blocks to form various double-layer structures, that is, structures that contain a double-layer β-sheet as the main structural part. An efficient computational strategy for constructing these double-layer arrangements from the equilibrated single-layer dimers was used to mimic the “steric zipper” arrangement of the peptide part as observed in some microcrystals [37–38], followed by an extensive relaxation MD run allowing the molecules to adjust their periodic arrangement to the best local minimum of free energy.

**Figure 13:**
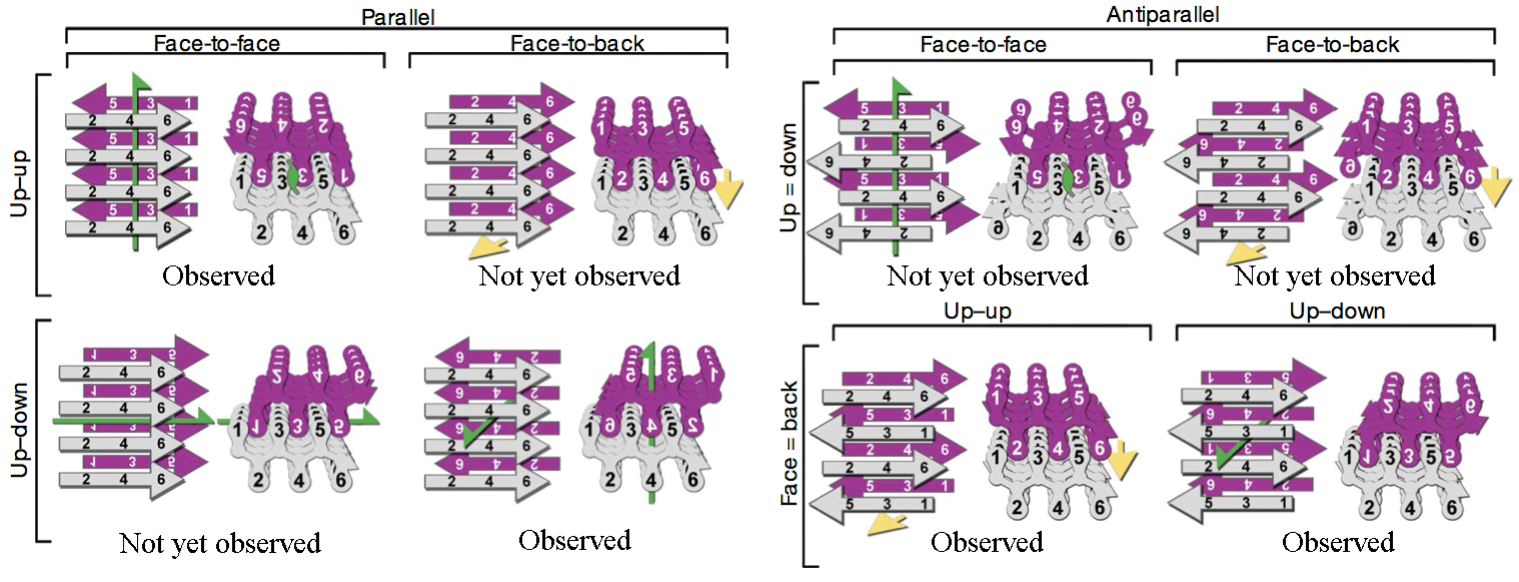
Possible options for the arrangement of β-sheets in a cross-β motif. Two identical sheets can be classified by the orientation of their faces (either “face-to-face” or “face-to- back”), the orientation of their strands (with both sheets having the same edge of the strand “up”, or one “up” and the other “down”), and whether the strands within the sheets are parallel or antiparallel. Both side views (left) and top views (right) show which of the six residues of the segment point into the zipper and which point outward. Green arrows show two-fold screw axes, and yellow arrows show translational symmetry. Below each class are listed protein segments that belong to that class. Reprinted by permission from Macmillan Publishers Ltd: Nature [37], copyright 2007.

The double-layer arrangements were then created from the single layer arrangements. In principle, possible hypothetical double tape arrangements of the peptide core are depicted schematically in Figure 13.

There are in total eight principal arrangement possibilities. However, due to experimental evidence and basic physical principles, many of them may be left out from further consideration. It is worth noting that the alternating nature of [Thr–Val]_3_ sequence exposes the hydrophilic threonine side chains from one side of the β-sheet and hydrophobic valine side chains from the other side. Thus one side of the β-sheet may be considered hydrophobic while the other hydrophilic. Assuming that the hydrophobic–hydrophilic interactions are one of the main driving forces, the structures with energetically unfavorable contacts of hydrophobic and hydrophilic moieties can be left out. This simple consideration already reduces the number of possible double-layer arrangements to the following three structures: The “face-to-face” antiparallel arrangement and two parallel face-to-face arrangements (“up-up” and “up-down” structures). The “up-down, face-to-face” parallel arrangement in turn is considered unlikely as (i) such a structural arrangement of β-strands is not observed experimentally to date for biological amyloid fibers, (ii) such an arrangement will not be favored by the interaction of dipole moments of the adjacent tapes, and (iii) moreover only one such arrangement is sterically allowed because of the bulkiness of the thiophene fragments of the hybrid molecule.

**Figure 14:**
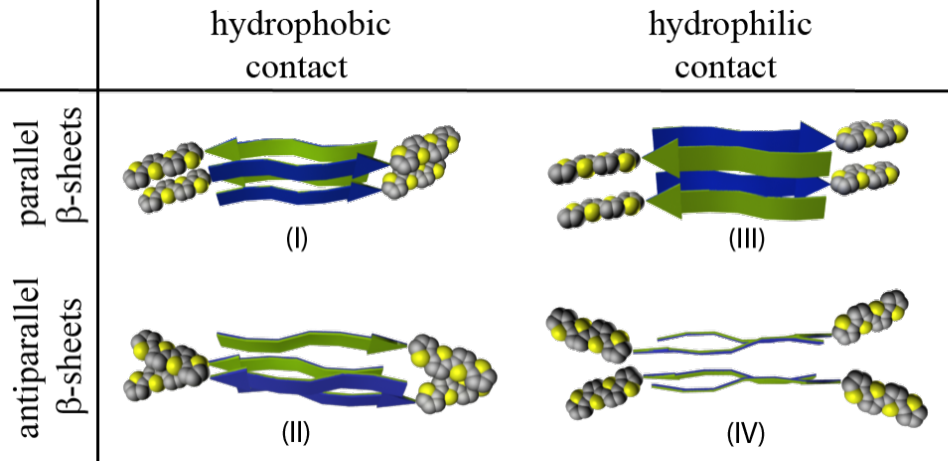
Schematic representations of constructed double-layer periodic arrangements from the hybrid molecule under study, classified by β-sheet orientation (parallel versus antiparallel) and the type of interlayer contact (hydrophobic versus hydrophilic) [23].

Each of the two remaining principal arrangements can be realized in two variants depending on which kind of faces (with exposed threonine or valine sidechains) are in contact. The resulting four arrangements are shown in Figure 14 (here and below they are denoted as I, II, III, and IV).

These structures were constructed as follows. First, the single-layer structure was replicated, turned 180° around the axis of the tape, and then adjusted in the lateral plane, so that the peptide segments of two single-layer fibrils would be approximately in register forming a “steric zipper” in the center of the fibril as seen in microcrystals of amyloidogenic peptides. Any overlap of molecular fragments that occurred (e.g., within alkylchains) was removed by small adjustments of the involved torsion angles, which would anyway reach their equilibrium values during the relaxation run. Since double-layers are considerably more complicated conformational assemblies, particularly with respect to the organization of the surfaces buried between the two β-sheets and the interaction of the side chains at these surfaces, a 10 ns MD relaxation run was performed for these periodic structures. During this run, a certain rearrangement of the side chain conformations between the β-sheets took place. The relative enthalpies of formation for these structures during the run were monitored, in order to track the system relaxation. The lowest relative enthalpy of formation was observed for structure III. Relative to structure III, structures I, II and IV have an additional enthalpy of formation of 11, 6 and 12 kcal/mol per molecule, respectively. Although this data set was obtained in vacuum simulations and the effect of solvent was neglected, it gives valuable quantitative data for the understanding of the hierarchy of interactions in such systems and is consistent with the supposition that hydrophilic interlayer contacts and an antiparallel arrangement of the β-sheets are the factors that lead to a gain in enthalpy of formation. However, in our case because of the specific geometry of the molecules, the aggregation pattern based on antiparallel β-sheets and hydrophilic interlayer contact (Figure 14, system IV) leads to the loss of close packing between the thiophene moieties and thus becomes energetically unfavorable.

Both obtained single-layer periodic arrangements and two of the double-layer structures with minimal enthalpy of formation (arrangements (II) and (III)) were used to construct long fibrils by replicating the periodic cell along the axis of the filament. For the selected systems, planar straight fibrils 80 β-strands in length (approximately 40 nm) were constructed and subjected to 10 ns MD simulations at *T* = 300 K. To this end, the LAMMPS simulation package [39], based on the domain decomposition strategy, was employed. The conformational evolution of the constructed aggregates during the simulations allows the study of the shape and morphology of the fibrils, as well as tracing the influence of the intermolecular arrangement on the conformation of the aggregates at the nanoscale. Comparative analysis of various fibrils (as well as comparison of the fibrils composed of hybrid molecules to those consisting only of peptides) allows us to discuss the role of various intermolecular interactions in stability and behavior of the aggregates, as well as to compare their geometry and behavior to the experimentally observed characteristics.

**Figure 15:**
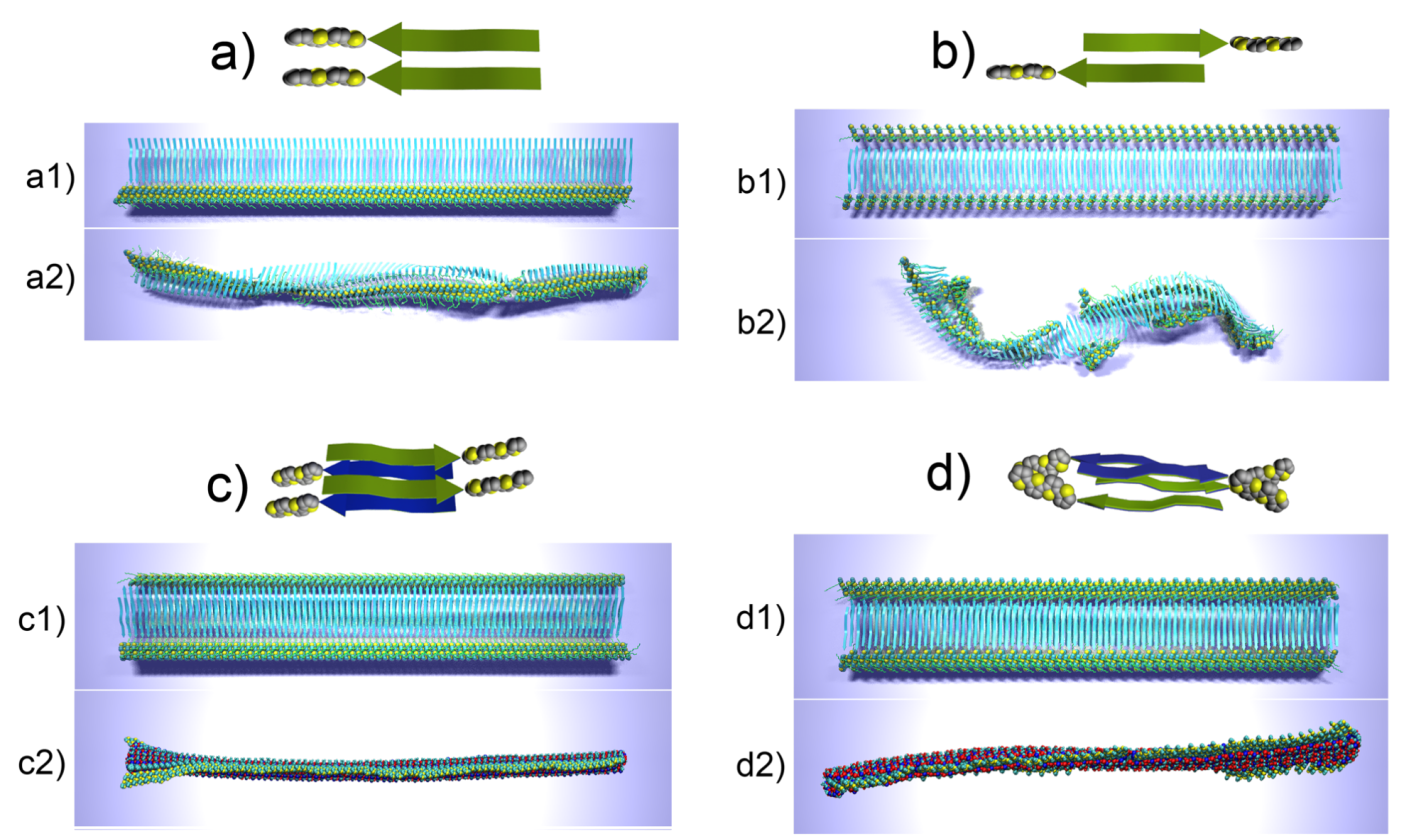
Snapshots of four different types of fibrils at the initial conformation (a1, b1, c1, d1) and after 10 ns of evolution (a2, b2, c2, d2). Peptide moieties are depicted with arrows, thiophene moiety using van der Waals spheres; images c2, c3, d2, d3 use van der Waals representation also for the peptide part for clarity [23].

Representative snapshots that describe the conformational evolution of different fibrillar arrangements are presented in Figure 15.

All the systems demonstrated their stability during the simulation run in the sense that all molecules preserved their relative positions in the aggregates with respect to their neighbors, however, certain conformational rearrangements both at the molecular level and at the nanoscale were observed. In all the cases, the β-sheet organization dominated the structure and remained the main scaffold for the fibril organization. A simulation time of 10 ns appeared to be enough to grasp the main characteristics of the fibril morphology and its evolution. As seen from Figure 15a and b, single layer fibrils are capable of a more pronounced conformational rearrangement than double layer fibrils, as the double-layer organization of the fibrils makes them stiffer and conformationally more stable. Further we will examine the evolution of different aggregate types.

The simulations of single layer fibrils based on the parallel arrangement of β-strands (Figure 15a), revealed that the fibril undergoes certain conformational changes during the evolution, however, it remains rather linear and axially rigid (Figure 15a2). The initially planar structure of the fibril undergoes certain twists with respect to its axis, while the conformation of the peptide backbone changes. As seen from Figure 15a2 the originally flat peptide sequences of the molecules have developed a kink by rotational rearrangement of the peptide backbone at the position of the glycine residue, which connects the [Thr–Val]_3_ sequence with the rest of the molecule. Since glycine has no side chain, it does not hinder the rotation around C–N and C–O bonds of the peptide backbone and hence allows the observed flexibility. Meanwhile, the thiophene moieties of the molecules remain in closely packed alignment, thus forming a continuously organized axial structure with a left-handed twist. On the contrary, the single layer fibril based on the antiparallel arrangement of β-strands, which lacks much of the thiophene–thiophene interactions (since the spacing between the thiophene segments is twice as large as in the case of the parallel arrangement), tends to curl to form a left-handed helical structure (Figure 15b). It should also be noted that the left-handed helix formed by a tape that has different types of faces (e.g., hydrophobic and hydrophilic face) may in principle exist in two conformations, that is, one where the hydrophobic side is directed to the inner compartment of the helix, and the reverse case. The variant of the helix curl should depend on the balance between the interactions of the side chains on each side of the tape with each other as well as with the solvent. Note that the fibril composed of the hybrid molecules (Figure 15b) tended to have the hydrophobic face of the tape at the inner part of the helix.

Contrary to the single layer fibrils, double layer fibrils result in almost planar tapes with a possible twist along the axis. The interactions between the adjacent single layer tapes strengthen the structure and molecular order by hydrogen bonds and steric interactions. However, the interactions also reduce the peptide flexibility, and thus do not favor additional peptide bending, which, e.g., led to the formation of kinks in the peptide backbones of single layer arrangements (Figure 15a). Moreover the stacking of two tapes with identical faces implies other “geometrical” considerations, such as suppression of helix formation, since each tape, if taken separately, would prefer to curl in the opposite direction to its neighbor, and thus the “curling” potential vanishes. The double tape fibril composed of parallel β-sheets stacked with hydrophilic faces (Figure 15c) remained surprisingly planar with only a small left-handed twist at one end of the fibril, which may be attributable to end effects.

Meanwhile the double tape arrangements based on antiparallel β-sheets (Figure 15d) formed a relatively planar, stable, left-handed twisted tape with an approximate twist of 30 degrees per 40 nm.

From the view point of various technological applications, the most important question concerns the arrangement of the conjugated thiophene moieties and the interplay between π−π stacking and β-sheet formation. As spacing between the molecules implied by the β-sheet structure corresponds to 4.8−5 Å, whereas the spacing between the planes of thiophene rings participating in π−π stacking interaction is estimated ideally to be around 3.3−3.5 Å [40–41], these interactions have competing behavior in terms of the periodicity implied during molecular aggregation. The analysis of histograms for the distribution of distances between the centers of mass of peptides and also between quaterthiophenes clearly reveals that the arrangement of the peptides into β-sheets dominates the periodicity implied for the aggregates for all types of simulated fibrils, thus meaning that the thiophene moieties have to adopt the periodic arrangement settled by the peptides. There are two possible variants of such behavior. In the case of a parallel arrangement of the β-strands, the thiophene moieties tilt synchronously, thus simultaneously preserving the high degree of ordering and fulfilling the periodicity restrictions implied by the β-sheets. In the case of the antiparallel arrangement, the proposed double layer fibril (Figure 15d) in principle has the same linear density of quaterthiophenes along the fibril as in the case of fibrils with parallel arrangement, because the thiophene moieties from different tapes come in contact to make a sort of “steric zipper” along each side of the double tape. However, in this case, the thiophene parts tend to lean towards one another to form dynamic clusters with gaps between them, which alleviate the differences between optimal inter-thiophene and inter-peptide distances.

The comparison of the conformational behavior (twisting or curling of the aggregates) of various simulated fibrillar aggregates and their spatial dimensions (cross section of 6−8 Å, heights of 1.4−1.8 Å (double layers)) with the available AFM data (Figure 7) suggests that the double layer arrangement based on parallel β-sheet organization (Figure 15c) fits well with the available experimental data and may be considered as the highly probable model for the actual aggregation pattern.

## Conclusion

Combining synthetic and biological building blocks to form various types of biomimetic thiophene-based hybrid compounds offers a means to construct new ordered supramolecular nanostructures and soft materials through self-organization, and such structures may exhibit a rich polymorphism over nanometer length-scales. The design of such hybrid molecules is a unique strategy, which aims to create smart nanomaterials that combine the properties of both the synthetic and biological components. Applying such an approach in the chemistry of conjugated compounds leads to new organizational concepts at nanoscales for these technologically important molecules.

This minireview was focused on and limited to a summary of recent advances in the understanding the self-organization processes observed for oligothiophene–oligopeptide conjugates, which are capable of forming long fibrillar aggregates in solution and on substrates. In particular, we reported on the successful synthesis of new bio-substituted organic semiconductor compounds where the rigid quaterthiophene block is either di- or monosubstituted with peptide sequences that display a high propensity to form β-sheets. These molecular hybrids can spontaneously self-assemble into stable fibrillar aggregates as visualized by AFM and TEM. We presented data on both kinked and nonkinked states of the peptide chains and found that the behavior of both symmetric (A–B–A) and asymmetric (A–B) hybrids in relation to the superstructure formation differs deeply, depending on the peptide conformation. These observations lead to the assumption that the mode of self-assembly of the individual molecules in their respective states, kinked and stretched, differs profoundly. Theoretical approaches based on quantum chemistry calculations and atomistic molecular dynamics (MD) simulations were attempted in order to reveal the possible intermolecular arrangements, their characteristic features, and to devise the possible arrangement models. Several models of the aggregation pattern of molecules for the A–B-and A–B–A-type conjugates were presented based on quantum chemical calculations. We have discussed a theoretical approach developed to study possible intermolecular arrangements and their characteristic features at finite temperature. This methodology incorporates available experimental data to suggest different variants of possible fibrillar nanostructures. These structural candidates were studied theoretically and their behavior was then cross-validated against available experimental evidence. Large-scale all-atom MD simulations for several proposed fibrillar models were also performed. They revealed the dependence of the fibrillar morphology on the intrinsic molecular organization of the fibrils. Using combined theoretical and experimental analysis, we have suggested the most likely models of fibril formation.

Future work should be aimed towards the development of new ways for the formation of semiconducting fiber-like supramolecular structures with mechanical properties mimicking those of natural materials such as silk or amyloid fibers. The idea is to obtain conductive materials with good mechanical strength and elasticity for various applications. Innovative methods such as computer simulations and combined multidisciplinary partnerships between chemists, physicists, and biochemists are required for the development and optimization of these bioinspired materials to provide fundamental understanding of their structural and charge transport properties.
